# Percent Fat Mass Is Inversely Associated With Bone Mass and Hip Geometry in Rural Chinese Adolescents

**DOI:** 10.1002/jbmr.40

**Published:** 2010-01-29

**Authors:** Xiumei Hong, Lester M Arguelles, Xin Liu, Hui-Ju Tsai, Yi-Hsiang Hsu, Binyan Wang, Shanchun Zhang, Zhiping Li, Gengfu Tang, Xue Liu, Jianhua Yang, Xiping Xu, Craig Langman, Xiaobin Wang

**Affiliations:** 1 Mary Ann and J Milburn Smith Child Health Research Program, Department of Pediatrics, Northwestern University Feinberg School of Medicine and Children's Memorial Hospital and Children's Memorial Research CenterChicago, IL, USA; 2 Center for Population Genetics, University of Illinois at Chicago School of Public HealthChicago, IL, USA; 3 Institute for Biomedicine, Anhui Medical UniversityHefei, People's Republic of China; 4 Hebrew SeniorLife Institute for Aging Research and Harvard Medical SchoolBoston, MA, USA; 5 Molecular and Integrative Physiological Sciences Program, Harvard School of Public HealthBoston, MA, USA; 6 Division of Kidney Diseases, Department of Pediatrics, Northwestern University Feinberg School of Medicine, Children's Memorial HospitalChicago, IL, USA; 7 Division of Biostatistics and Bioinformatics, Institute of Population Health Sciences, National Health Research InstitutesZhunan, Taiwan

**Keywords:** percent fat mass, hip geometry, bone mineral content, adolescence, coheritability

## Abstract

This study was an attempt to examine the phenotypic, genetic, and environmental correlations between percent fat mass (PFM) and bone parameters, especially hip geometry, among 786 males and 618 females aged 13 to 21 years from a Chinese twin cohort. PFM, bone area (BA), bone mineral content (BMC), cross-sectional area (CSA), and section modulus (SM) were obtained by dual-energy X-ray absorptiometry. Multiple linear regression models were used to assess the PFM-bone relationships. A structural equation model for twin design was used to estimate genetic/environmental influences on individual phenotype and phenotypic correlations. After controlling for body weight and other pertinent covariates, we observed inverse associations between PFM and bone parameters: Compared with the lowest age- and gender-specific tertile of PFM, males in the highest tertile of PFM had lower measures of whole-body-less-head BA (WB-BA), lumbar spine BA (L_2_–L_4_-BA), total-hip BA (TH-BA), total-hip BMC, CSA, and SM (*p* < .005 for all, adjusted *p* < .05). Similar inverse associations were observed in females for all the preceding parameters except WB-BA and L2–L_4_-BA. These associations did not vary significantly by Tanner stages. In both genders, the estimated heritabilities were 80% to 86% for BMC, 67% to 80% for BA, 74% to 77% for CSA, and 64% for SM. Both shared genetics and environmental factors contributed to the inverse PFM-bone correlations. We conclude that in this sample of relatively lean Chinese adolescents, at a given body weight, PFM is inversely associated with BA, BMC, and hip geometry in both genders, and such associations are attributed to both shared genetic and environmental factors. © 2010 American Society for Bone and Mineral Research.

## Introduction

The increasing prevalence of obesity and its health consequences have become a major public health challenge worldwide. Obesity is a well-established risk factor for excess cardiovascular disease, stroke, and type 2 diabetes in both adolescents and adults.(1,2) Previously, we observed that percent fat mass (PFM), an index of obesity, was inversely related to bone mineral density (BMD) and the risk of osteoporotic fractures in an adult populations,(3) which has also been observed in some other but not all studies.(4) There is still an important knowledge gap on the association between fat mass and an array of bone parameters such as bone mass, bone area, and hip geometry, especially among adolescents.

Delineating the association between fat mass and bone parameters in adolescents is important but particularly challenging. In contrast to mature adulthood, adolescence is a period of rapid physical growth and functional maturation. Adolescence is also a critical stage for bone growth, including bone length, width, area, material, and geometry. Factors or conditions that alter bone formation or enhance bone resorption during adolescence will lead to suboptimal bone growth, presumably putting the person at greater risk of osteoporotic fracture later in life.(5) Methodologically, one has to take into account the normal growth and development of fat mass during adolescence and to tease out the positive mechanical loading effect of body weight on bone from the non-weight-bearing effect of fat mass. One also has to be able to adequately account for many other factors that may affect bone parameters, such as age,(6) gender,(6) Tanner stage,(6) and physical activity.(7) Furthermore, the type of bone varies by skeletal region of the body (ie, vertebra, hip, etc.), and both bone material properties and bone geometry are independent determinants of bone strength. It has been suggested that changes in bone geometry can occur that may affect bone mechanical strength but will not necessarily be apparent in bone material.(8) Thus the association between fat mass and bone parameters may vary by skeletal regions and/or by specific bone parameters (ie, bone material versus bone geometry) being studied.

Longitudinal studies in childhood and adolescence have shown that higher body weight is a strong predictor of higher bone mass later in life.(9) However, the associations between body weight and bone parameters may not necessarily represent the correlation between fat mass and bone. Body weight consists of lean mass, fat mass, and bone mass. It is commonly believed that lean mass mediates the positive effect of body weight on bone mass.(10) In contrast, fat tissue is a metabolically active organ, and thus it may influence the skeleton not only through the weight-bearing pathways but also through the non-weight-bearing pathways, including the hormonal metabolism of adipocytes. To date, only a few studies have explored the associations between fat mass (or obesity) and bone in adolescents, and they yielded inconclusive results.(11–18) One important reason is that some of the available studies did not take into account the positive mechanical loading effect of body weight on bone parameters. Of note, most of these previous studies focused on bone area and bone mass,(11,12,16–18) whereas limited data are available on bone strength or hip geometry,(13–15) although the latter has been recognized as an important factor for hip fracture.(19) Furthermore, few studies among adolescents have explored the effects of Tanner stage on the fat mass–bone relationship. Finally, fat mass and bone parameters are complex traits that are influenced by environmental factors, genetic factors, and their interactions. Currently, there are few data examining the genetic influence on the relationship between fat mass and bone parameters.

This study sought to examine the association of PFM with an array of bone parameters, including bone area (BA) and bone mineral content (BMC), at different skeletal regions, as well as two hip geometry indices at the femoral neck region, in rural Chinese adolescents after controlling for the mechanical loading effect of body weight, Tanner stage, and other pertinent covariates. We also explored whether the PFM-bone associations vary by gender, Tanner stage, bone parameters, and skeletal region. Additionally, we estimated to what degree the PFM-bone associations were contributed by the shared genetic factors using a twin design.

## Methods

### Study population and procedures

The study populations were part of a community-based prospective twin cohort that was recruited during 1998–2000 in the rural area of Anqing, China (baseline study). Since 2005, twins who participated in the baseline study have been followed, including clinical measurement of height, weight, body composition, and bone mass using the same study protocols as in the baseline. Detailed information on enrollment criteria of the twins at the baseline and follow-up studies was described previously.(20) This study was approved by the institutional review boards of Children's Memorial Hospital and the University of Illinois at Chicago and the Ethics Committee of Anhui Medical University. Written informed consent was obtained from each participant. In this report we used data collected at follow-up for subjects aged 13 to 21 years, which met the definition of adolescence by the American Academy of Pediatrics (http://www.aap.org/healthtopics/stages.cfm).

A comprehensive questionnaire was used to collect each participant's demographic, occupational, and lifestyle information, as well as dietary information. The short version of the International Physical Activity Questionnaire (IPAQ Short) (http://www.ipaq.ki.se) was applied to evaluate physical activity level using the “last 7 days” as a reference period. Detailed definition for low, moderate, or high physical activity was described previously.(20) In brief, each type of activity was weighted by its metabolic-equivalent (MET) level, and a score in MET-minutes (MET-min) was produced for each subject. “High” physical activity level was defined as (1) vigorous-intensity activity on 3 or more days and accumulating 1500 or more MET-min/week, or (2) 7 or more days of any combination of walking, moderate-intensity, or vigorous-intensity activities, achieving 3000 or more MET-min/week. “Moderate” physical activity level was defined as meeting any one of the following criteria: (1) 3 or more days of vigorous-intensity activity for 20 minutes/day or more, (2) 5 or more days of moderate-intensity activity or walking for at least 30 minutes/day, or (3) 5 or more days of any combination of walking or moderate- or vigorous-intensity activities achieving 600 or more MET-min/week. Individuals who did not meet criteria for high or moderate physical activity were considered to have a “low” physical activity level.

### Anthropometry

Height was measured without shoes to the nearest 0.1 cm on a portable calibrated stadiometer. Similarly, weight was measured without shoes to the nearest 0.1 kg with the subject standing motionless in the center of a calibrated scale. In addition, Tanner stage (I through V) was determined by visual inspection by a trained physician.(21,22)

### Measurement of BMC, BA, and body composition

Dual-energy X-ray absorptiometry (DXA; GE-Lunar Prodigy, Waukesha, WI, USA, with enCORE 6.0 software) was used to measure soft tissue body composition, BMC (in grams), and BA (in square centimeters) through whole-body, lumbar spine, and total-hip scans. All the bone densitometry readings were performed in a single study center in China by an experienced technologist who received training from the manufacturer. The machine was calibrated daily with a phantom. Seventy-one individuals have been measured repeatedly at different skeletal sites. The coefficient of variability (*CV*%) is 1.3% at the whole body and 2.1% at the total hip, respectively. Whole-body fat mass (FM) and lean mass (LM) were expressed in terms of weight (kilograms). PFM was calculated as FM × 100/weight in kilograms.

### Hip structure analysis (HSA)

With the manufacturer's HSA program that is commercially available, hip geometry variables were calculated automatically from the scan image and bone distribution variables derived from information contained within DXA X-ray absorption curves.(23) In this study, two variables were studied: (1) cross-sectional area (CSA, square centimeters) of the minimum cross-sectional moment of inertia (CSMI) section within the femoral neck region and (2) section modulus (SM, cubic centimeters), which was calculated as the minimum CSMI within the femoral neck region divided by distance from the center of mass to the superior neck margin for the section of minimum CSA.

### Zygosity identification

Twin zygosity was determined using DNA fingerprint technology by genotyping 10 microsatellite markers with high heterozygosity (>70%) and located on different autochromosomes.(24)

### Statistical analysis

The primary outcomes were whole-body-less-head BA (WB-BA) and BMC (WB-BMC), lumbar-spine BA (L_2_–L_4_-BA) and BMC (L_2_–L_4_-BMC), total-hip BA (TH-BA) and BMC (TH-BMC), and two hip geometric indices, CSA and SM. During the analyses, each BMC was adjusted for the corresponding BA by including it in the linear regression model. We excluded outliers (*n* = 44) that were 4 SD away from the respective average obtained from the analyses. All the analyses were conducted by gender in the remaining 1404 subjects.

First, we plotted the relationships between PFM and each bone parameter, adjusting for the potentially confounding effects of age, Tanner stage, weight, height, menarche status (for females only), physical activity, passive or active smoking (yes/no), and occupation. For BMCs, the corresponding BA also was adjusted. Second, the gender- and age-specific tertiles of PFM was created by tertiling PFM within each 1-year strata of age in males and females, separately. Multiple linear regressions, with adjustment of the pertinent covariates, were used to assess the associations of PFM tertiles with each of the bone parameters. To examine the influence of Tanner stage on the PFM-bone associations, these regression models were stratified by Tanner stage, and the interaction effect between PFM tertiles and Tanner stage was tested by including a product term in the model. Generalized estimating equations (GEEs) were used in all models to accommodate intrapair correlations. SAS Version 9.1 (SAS Institute, Cary, NC, USA) was used for all analyses. Further, the Bonferroni correction, with the significant level α = 0.00625 (= 0.05/8 phenotypes), was applied to control for multiple tests.

Structural equation modeling was applied to estimate the additive genetic component (*a*^2^) and shared (*c*^2^) and individual-specific (*e*^2^) environmental components for the phenotypic variance using the twin design. We evaluated three different models, including a model encompassing additive genetic influences (A), common (C), and individual-specific (E) environment influences (the full ACE model), a model encompassing A and E (AE model), and a model encompassing C and E (CE model). Under the principle of parsimony, the best-fitting model was defined as the one not having a significantly worse fit compared with the full ACE model (ie, the chi-square test was not significant with *p* > .05). To estimate the genetic/environmental influence on the phenotypic correlations between PFM and bone parameters, the best-fitted bivariate Cholesky decomposition model, defined based on the same criteria mentioned earlier, was applied to calculate genetic (*r*_G_), shared environmental (*r*_C_), and individual-specific environmental correlations (*r*_E_) between each pair of phenotypes. Then the genetic (*C*_GCP_), common (*C*_CCP_), and individual-specific (*C*_ECP_) environmental contributions to the phenotypic correlations were calculated as *r*_G_ × 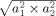, *r*_C_ × 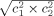, and *r*_e_ × 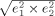, respectively. To adjust for important confounding factors, PFM and bone parameters were modeled on age, Tanner stage, weight, height, menarche status (for females only), physical activity, passive or active smoking (yes/no), occupation, and the corresponding BA (for all the BMC measures only), and the residuals from these models were used to estimate the genetic/environmental contribution to the phenotypic variations as well as their correlations using the Mx software (http://www.vcu.edu//mx/).(25)

## Results

### Epidemiologic and clinical characteristics

A total of 786 males and 618 females, from a same-sex twin cohort, with a mean age of 16.6 ± 2.0 years, were included in this study. About 84.0% of the subjects had available zygosity information: In males, there were 200 monozygotic (MZ) and 128 dizygotic (DZ) pairs, and in females, there were 179 MZ and 83 DZ pairs. The epidemiologic characteristics of the study population are shown in Table 1. Males had significantly higher values in height, weight, lean mass, BA and BMC at different skeletal sites, CSA and SM (*p* < .0001) at the femoral neck, and a higher percentage of high physical activity. However, PFM in males (mean ± SD 11.5% ± 4.9%) was less than half that in females (27.2% ± 5.6%). In general, our study subjects, especially males, were relatively lean compared with a Western population.

**Table 1:** Characteristics of 1404 Chinese Adolescents from the Anqing Twin Cohort (Mean ± SD)

Variable	Male (*n* = 786)	Female (*n* = 618)	*p* Value^a^
Age, years	16.6 ± 2.0	16.6 ± 2.0	.750
Weight, kg	48.7 ± 8.1	46.1 ± 6.5	<.001
Height, cm	160.7 ± 7.9	152.6 ± 5.3	<.001
Body composition, kg			
Whole-body fat mass	5.7 ± 3.2	12.8 ± 4.0	<.001
Total lean mass	41.5 ± 6.5	31.6 ± 3.2	<.001
Percent fat mass (%)	11.5 ± 4.9	27.2 ± 5.6	<.001
Bone area (BA), cm^2^			
Whole-body-less-head	1659.5 ± 248.2	1517.0 ± 179.1	<.001
Lumbar spine	39.0 ± 5.7	35.7 ± 3.8	<.001
Total hip	31.0 ± 3.3	27.0 ± 2.0	<.001
Bone mineral content (BMC), g			
Whole-body-less-head	1505.6 ± 357.3	1319.3 ± 230.3	<.001
Lumbar spine	37.2 ± 9.9	35.6 ± 6.7	<.001
Total hip	28.4 ± 5.8	23.9 ± 3.5	<.001
Hip geometry indices			
Cross-sectional area, cm^2^	1.4 ± 0.3	1.2 ± 0.2	<.001
Section modulus, cm^3^	0.55 ± 0.14	0.42 ± 0.08	<.001

	*n* (%)	*n* (%)	
Physical activity			
Low	234 (29.8)	237 (38.3)	
Moderate	278 (35.4)	207 (33.5)	
High	191 (24.3)	105 (17.0)	
Unkown	83 (10.5)	69 (11.2)	<.001
Tanner stage			
I	65 (8.2)	9 (1.4)	
II	116 (14.8)	98 (15.9)	
III	154 (19.6)	235 (38.0)	
IV	161 (20.5)	160 (25.9)	
V	155 (19.7)	103 (16.7)	
Unknown	135 (17.2)	13 (2.1)	<.001
Passive smoking, yes	551 (70.1)	400 (64.7)	.060
Current smoker	77 (9.9)	0	<.001
Occupation, student	580 (73.8)	405(65.5)	<.001
Menarche, yes	—	550 (89.0)	

a A *t* test was performed to compare the difference of continuous variables, and a chi-square test was performed to compare categorical variables between males and females, respectively.

### Attained level of hip geometry variables by age and Tanner stage

**Fig. 1:**
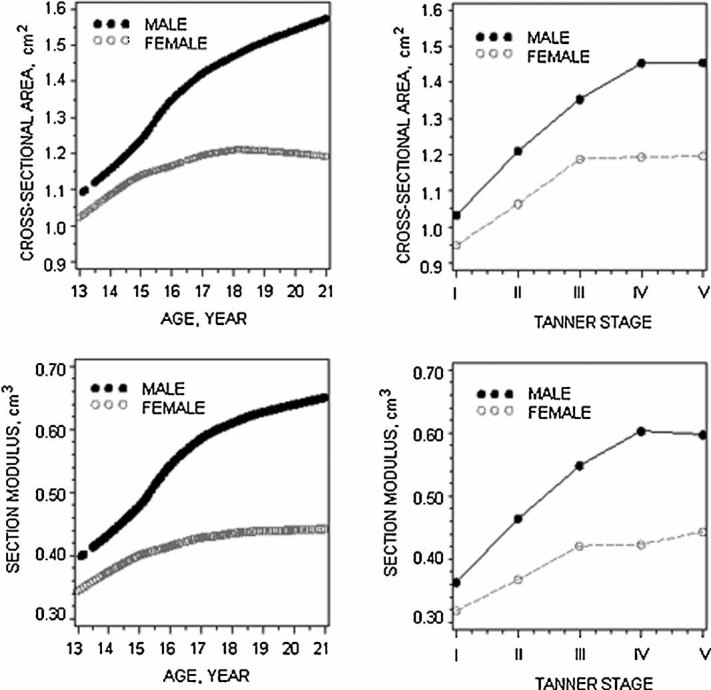
The attained level of hip geometry variables by age and Tanner stage among 1404 Chinese adolescents from the Anqing twin cohort.

Previously, we reported gender differences in BMC/BA by either age or Tanner stage in a subset of this cohort.(6) We observed a similar trend for CSA and SM in this study. As shown in Fig. 1, CSA and SM increased with age linearly until approximately 17 years of age (or Tanner stage IV) in males or approximately 15 years of age (or Tanner stage III) in females and slowed thereafter. From the age of 13, there was an increasing gender difference in CSA and SM, with CSA and SM significantly higher in males than in females across Tanner stages II through V.

### Relationship between BMC and hip geometry

We examined the relationship of BMC with hip geometric variables after adjusting for age, Tanner stage, weight, height, menarche status (for females only), physical activity, passive or active smoking, occupation, and the corresponding BA. We found that BMC at the whole body, lumbar spine, and total hip was significantly and positively associated with CSA and SM (*p* < .0001) in males and females, respectively (Supplemental Table S1). These positive associations remained unchanged when age- and gender-specific tertile of PFM was further adjusted in the model.

### Association of PFM with bone parameters

**Fig. 2:**
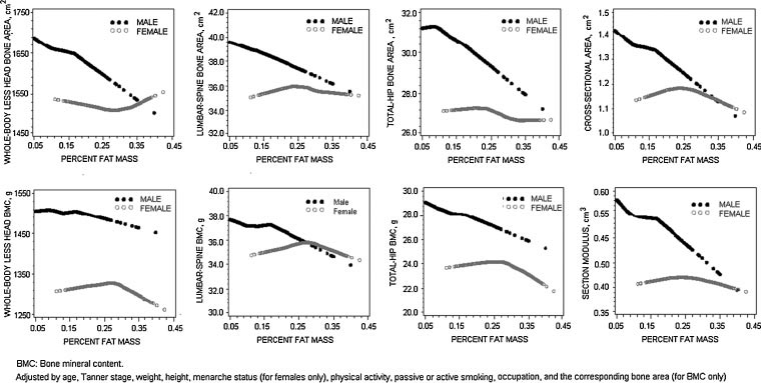
The gender-specific relationship of percent fat mass with an array of adjusted bone parameters among 1404 Chinese adolescents from the Anqing twin cohort.

Each bone parameter, after adjustment for age, Tanner stage, weight, height, physical activity, menarche status, active or passive smoking, and occupation, was plotted against PFM by gender (Fig. 2). In males, WB-BA, L_2_–L_4_-BA, TH-BA, L_2_–L_4_-BMC, TH-BMC, CSA, and SM tended to decrease with increasing PFM; in females, bone parameters do not appear to change with increasing PFM when PFM < 0.25. However, for PFM ≥ 0.25, all bone parameters, except for WB-BA and L_2_–L_4_-BA, decreased with increasing PFM.

Table 2 summarizes the associations between age- and gender-specific tertiles of PFM and each bone parameter. In males, the crude mean values for L_2_–L_4_-BA and TH-BA were the lowest in the top tertile of PFM, whereas the crude mean values for the other bone parameters did not differ significantly across PFM tertiles. However, after adjustment for body weight and the other covariates, those in the top tertile of PFM had significantly lower WB-BA (β ± SE −39.3 ± 9.0 cm^2^, *p* < .0001), lower L_2_–L_4_-BA (−1.34 ± 0.32 cm^2^, *p* < .0001), lower TH-BA (−1.14 ± 0.20 cm^2^, *p* < .0001), lower TH-BMC (−1.06 ± 0.34 g, *p* = .002), lower CSA (−0.08 ± 0.02 cm^2^, *p* < .0001), and lower SM (−0.04 ± 0.01 cm^3^, *p* = .0004) than those in the bottom PFM tertile. No significant relationships between PFM and WB-BMC or L_2_–L_4_-BMC were observed in males.

**Table 2:** Associations of Age- and Gender-Specific Tertile of Percent Fat Mass (PFM) With Bone Parameters in 1404 Chinese Adolescents From the Anqing Twin Cohort

		Male			Female			Total population		
				
Phenotype	PFM Tertile	Mean ± SD	β ± SE^a^	*p* Value^a^	Mean ± SD	β ± SE^a^	*p* Value^a^	Mean ± SD	β ± SE^a^^b^	*p* Value^a^^b^
WB-BA	Low	1659.1 ± 219.1	ref		1447.1 ± 169.2	ref		1566.1 ± 224.8	ref	
	Middle	1663.4 ± 259.6	−12.7 ± 7.0	.072	1505.2 ± 164.7	−17.2 ± 7.7	.025	1593.5 ± 236.0	−13.4 ± 5.3	.011
	High	1655.9 ± 263.9	−39.3 ± 9.0	<.0001^c^	1598.5 ± 170.7	−9.9 ± 10.0	.322	1630.6 ± 229.2	−25.4 ± 6.9	.0003^c^
L_2_–L_4_-BA	Low	39.5 ± 5.2	ref		34.9 ± 4.0	ref		37.5 ± 5.3	ref	
	Middle	39.3 ± 5.9	−0.23 ± 0.26	.379	35.8 ± 3.6	−0.11 ± 0.27	.688	37.8 ± 5.3	−0.22 ± 0.19	.239
	High	38.1 ± 5.9	−1.34 ± 0.32	<.0001^c^	36.5 ± 3.6	−0.25 ± 0.35	.463	37.4 ± 5.1	−0.99 ± 0.24	<.0001^c^
TH-BA	Low	31.3 ± 2.9	ref		26.9 ± 1.9	ref		29.4 ± 3.3	ref	
	Middle	31.3 ± 3.6	−0.15 ± 0.16	.354	27.0 ± 2.0	−0.46 ± 0.17	.006^c^	29.4 ± 3.7	−0.33 ± 0.12	.005^c^
	High	30.4 ± 3.4	−1.14 ± 0.20	<.0001^c^	27.2 ± 2.0	−1.00 ± 0.21	<.0001^c^	29.0 ± 3.3	−1.14 ± 0.15	<.0001^c^
WB-BMC	Low	1496.5 ± 318.9	ref		1233.4 ± 227.6	ref		1381.1 ± 311.0	ref	
	Middle	1507.2 ± 375.6	−1.2 ± 8.0	.882	1312.1 ± 219.5	−0.6 ± 7.3	.933	1421.0 ± 330.5	−2.1 ± 5.7	.707
	High	1513.1 ± 375.2	−8.6 ± 10.0	.394	1411.3 ± 209.4	−31.2 ± 9.9	.002^c^	1468.3 ± 317.0	−21.4 ± 7.2	.003^c^
L_2_–L_4_-BMC	Low	37.7 ± 9.3	ref		33.7 ± 7.0	ref		36.0 ± 8.6	ref	
	Middle	37.3 ± 10.2	−0.46 ± 0.33	.173	35.8 ± 7.0	−0.01 ± 0.36	.969	36.6 ± 9.0	−0.19 ± 0.25	.439
	High	36.5 ± 10.2	−0.35 ± 0.44	.421	37.2 ± 5.6	−0.54 ± 0.46	.239	36.8 ± 8.5	−0.32 ± 0.32	.327
TH-BMC	Low	28.6 ± 5.2	ref		23.3 ± 3.5	ref		26.3 ± 5.2	ref	
	Middle	28.6 ± 6.3	−0.42 ± 0.29	.148	24.0 ± 3.7	−0.55 ± 0.28	.052	26.5 ± 5.8	−0.38 ± 0.21	.071
	High	27.9 ± 5.8	−1.06 ± 0.34	.002^c^	24.4 ± 3.1	−1.76 ± 0.37	<.0001^c^	26.4 ± 5.1	−1.24 ± 0.26	<.0001^c^
CSA	Low	1.38 ± 0.24	ref		1.13 ± 0.16	ref		1.27 ± 0.24	ref	
	Middle	1.35 ± 0.27	−0.05 ± 0.02	.001^c^	1.17 ± 0.16	−0.02 ± 0.01	.073	1.27 ± 0.25	−0.04 ± 0.01	.0003^c^
	High	1.35 ± 0.25	−0.08 ± 0.02	<.0001^c^	1.20 ± 0.15	−0.08 ± 0.02	<.0001^c^	1.28 ± 0.23	−0.08 ± 0.01	<.0001^c^
SM	Low	0.56 ± 0.13	ref		0.40 ± 0.08	ref		0.49 ± 0.13	ref	
	Middle	0.55 ± 0.15	−0.02 ± 0.01	.030	0.41 ± 0.08	−0.01 ± 0.01	.152	0.49 ± 0.14	−0.02 ± 0.01	.004^c^
	High	0.54 ± 0.14	−0.04 ± 0.01	.0004^c^	0.44 ± 0.08	−0.02 ± 0.01	.005^c^	0.49 ± 0.13	−0.03 ± 0.01	<.0001^c^

WB-BA = whole-body-less-head bone area (BA); L_2_–L_4_-BA = lumbar-spine BA, TH-BA = total-hip BA; WB-BMC = whole-body-less-head bone mineral content (BMC); L_2_–L_4_-BMC = lumbar-spine BMC; TH-BMC = total-hip BMC; CSA = cross-sectional area; SM = section modulus.

a The model adjusted for age, Tanner stage, weight, height, menarche status (for females only), physical activity, passive or active smoking, occupation, and the corresponding bone area (for BMC only).

b Gender also was included in the model.

c Adjusted *p* < .05 after the Bonferroni correction.

In females, the crude mean values for each bone parameter were the highest in the top tertile of PFM, but after adjustment for the covariates, those in the top tertile of PFM had significantly lower TH-BA (β ± SE −1.00 ± 0.21, *p* < .0001), WB-BMC (−31.2 ± 9.9, *p* = .002), TH-BMC (−1.76 ± 0.37, *p* < .0001), CSA (−0.08 ± 0.02, *p* < .0001), and SM (−0.02 ± 0.01, *p* = .005) than those in the bottom tertile. The relationships between PFM and the other bone parameters, including WB-BA, L_2_–L_4_-BA, and L_2_–L_4_-BMC, were insignificant.

We performed the same analyses in the total population and found similar inverse relationships between PFM tertiles and each bone parameter (Table 2). The inverse associations in males, females, and the total population remained significant after the Bonferroni correction (Table 2). Additional analyses of age- and gender-specific tertiles of fat mass in relation to bone parameters showed that their associations were comparable with the associations between PFM tertiles and bone parameters (data not shown). We also repeated our analyses by removing 99 subjects older than 19 years of age (56 males and 43 females) who did not meet the World Health Organization (WHO) definition of adolescence and found that the inverse relationship between PFM and each bone parameter remained unchanged (data not shown).

### Effect of Tanner stage on the PFM-bone associations

To examine the effect of Tanner stage on the association between PFM tertiles and bone measures, least-squares means and standard errors of TH-BMC, TH-BA, CSA, and SM across PFM tertiles in each Tanner stage, after adjustment of age, weight, height, physical activity, menarche status, active or passive smoking, and occupation, are presented in Supplemental Figures S1 and S2. In males, those in the higher tertile group had lower TH-BMC values in Tanner stages I, IV, and V and had lower TH-BA, CSA, and SM values in each Tanner stage, with the strongest associations in Tanner stages IV and V. No significant interactive effect was detected between PFM and Tanner stage on these four phenotypes. In females, Tanner stage I was excluded from this subset of analyses owing to very limited sample size (*n* = 9). In Tanner stage II and above, females in the higher tertile group of PFM also had lower TH-BMC values in Tanner stages II through IV and had lower TH-BA, CSA, and SM values in all Tanner stages, with the strongest associations in Tanner stages III and IV. A modest interactive effect between PFM and Tanner stage was observed on CSA only (*p* = .045), and it became insignificant after the Bonferroni correction. In males, patterns similar to TH-BA were found for WB-BA and L_2_–L_4_-BA, and in females, a pattern similar to TH-BMC was observed for WB-BMC (data not shown).

### Genetic and environmental contribution to the PFM-bone association

Table 3 summarizes the univariate structural equation modeling results for each trait in the subset of samples with available zygosity information. It is important to note that the AE model was the best-fitting model for all the bone parameters and PFM (Supplemental Table S2). From the AE model, the estimated heritability for BA and BMC at different skeletal sites ranged from 67% to 80% and 80% to 86%, respectively, in both genders. The estimated heritability for CSA was 74% in males and 77% in females. The estimated heritability for SM was 64% for both genders.

**Table 3:** Heritability Estimates^a^ of Bone Parameters and Percent Fat Mass in 590 Same-Sex Chinese Twins From the Anqing Twin Cohort

	Male(200 MZ and 128 DZ pairs)		Female (179 MZ and 83 DZ pairs)	
		
Outcome^b^	*a*^2^ (95% CI)^c^	*e*^2^ (95% CI)^c^	*a*^2^ (95% CI)^c^	*e*^2^ (95% CI)^c^
WB-BA	0.80 (0.74–0.84)	0.20 (0.16–0.26)	0.74 (0.67–0.79)	0.26 (0.21–0.33)
L_2_–L_4_-BA	0.76 (0.69–0.80)	0.24 (0.20–0.31)	0.67 (0.60–0.74)	0.33 (0.26–0.40)
TH-BA	0.71 (0.64–0.77)	0.29 (0.23–0.35)	0.80 (0.74–0.84)	0.20 (0.16–0.26)
WB-BMC	0.83 (0.78–0.86)	0.17 (0.14–0.22)	0.80 (0.75–0.84)	0.20 (0.16–0.25)
L_2_–L_4_-BMC	0.86 (0.83–0.89)	0.14 (0.11–0.17)	0.85 (0.80–0.88)	0.15 (0.12–0.20)
TH-BMC	0.81 (0.76–0.85)	0.19 (0.15–0.24)	0.85 (0.80–0.88)	0.15 (0.12–0.20)
Cross-sectional area	0.74 (0.68–0.79)	0.26 (0.21–0.32)	0.77 (0.71–0.82)	0.23 (0.18–0.29)
Section modulus	0.64 (0.56–0.71)	0.36 (0.29–0.44)	0.64 (0.55–0.71)	0.36 (0.29–0.45)
Percent fat mass	0.83 (0.78–0.86)	0.17 (0.14–0.22)	0.77 (0.71–0.82)	0.23 (0.18–0.29)

WB-BA = whole-body-less-head bone area (BA); L_2_–L_4_-BA = lumbar spine BA, TH-BA = total-hip BA; WB-BMC = whole-body-less-head bone mineral content (BMC); L_2_–L_4_-BMC = lumbar spine BMC; TH-BMC = total-hip BMC.

a Heritability (*a*^2^) was estimated using the best-fit AE model.

b Adjusted for age, Tanner stage, weight, height, menarche status, physical activity, passive or active smoking, occupation, and the corresponding bone area (for BMC only).

c 95% confidence intervals (CI) were calculated for each parameter estimate. These parameters were statistically insignificant if their CIs include 0.

Table 4 presents the estimation of genetic/environmental correlations between PFM and bone parameters observed in Table 2. The best-fitting AE bivariate model was applied for all the trait pairs. In males, the genetic correlations (*r*_G_) and the individual-specific environmental correlations (*r*_E_) for each PFM-bone pair were negative and significant. The proportions of phenotypic correlations between PFM and various bone parameters explained by shared genetics were as follows: 87% (= −0.27/−0.31) for WB-BA, 70% for L_2_–L_4_-BA, 92% for TH-BA, 76% for TH-BMC, 84% for CSA, and 84% for SM. The rest of these phenotypic correlations were explained by individual-specific environmental factors. Similarly, in females, we found that the phenotypic correlations between PFM and the five bone parameters (including TH-BA, WB-BMC, TH-BMC, CSA, and SM) could be explained by both shared genetics and individual-specific environmental factors, although the genetic correlation (*r*_G_) for the PFM–WB-BMC pair, as well as individual-specific environmental correlations (*r*_E_) for the PFM-CSA and PFM-SM pairs, did not attain statistical significance (Table 4).

**Table 4:** Genetic and Environmental Influence to the Phenotypic Correlations Between Percent Fat Mass^a^ and Bone Parameters^a^ in 590 Same-Sex Chinese Twins From the Anqing Twin Cohort

	Variance components correlations^b^		Genetic/environmental contribution^c^	
		
Bone parameter^a^	*r*_G_ (95% CI)	*r*_E_(95% CI)	*C*_GCP_	*C*_ECP_
Male				
WB-BA	−0.33 (−0.43, −0.22)	−0.21 (−0.33, −0.07)	−0.27	−0.04
L_2_–L_4_-BA	−0.25 (−0.35, −0.14)	−0.27 (−0.48, −0.24)	−0.19	−0.08
TH-BA	−0.44 (−0.53, −0.34)	−0.14 (−0.27, −0.01)	−0.34	−0.03
TH-BMC	−0.19 (−0.29, −0.08)	−0.25 (−0.28, −0.12)	−0.16	−0.05
Cross-sectional area	−0.33 (−0.43, −0.22)	−0.22 (−0.35, −0.09)	−0.26	−0.05
Sectional modulus	−0.29 (−0.40, −0.17)	−0.17 (−0.30, −0.04)	−0.21	−0.04
Female				
TH-BA	−0.23 (−0.34, −0.10)	−0.33 (−0.45, −0.20)	−0.18	−0.07
WB-BMC	−0.09 (−0.21, 0.05)	−0.32 (−0.44, −0.19)	−0.06	−0.07
TH-BMC	−0.15 (−0.27, −0.02)	−0.20 (−0.34, −0.06)	−0.12	−0.04
Cross-sectional area	−0.17 (−0.20, −0.04)	−0.11 (−0.25, 0.03)	−0.13	−0.03
Sectional modulus	−0.15 (−0.28, −0.01)	−0.08 (−0.21, 0.06)	−0.10	−0.02

WB-BA = whole-body-less-head bone area (BA); L_2_–L_4_-BA = lumbar spine BA; TH-BA = total-hip BA; WB-BMC = whole-body-less-head bone mineral content (BMC); L_2_–L_4_-BMC = lumbar spine BMC; TH-BMC = tota-hip BMC.

a Adjusted for age, Tanner stage, weight, height, menarche status, physical activity, passive or active smoking, occupation, and the corresponding bone area (for BMC only).

b *r*_G =_ genetic correlation between paired traits; *r*_E =_ individual-specific environmental correlation between paired traits. These parameters were statistically insignificant if their CIs included 0.

c *C*_GCP =_ genetic contribution to phenotypic correlations; *C*_ECP =_ individual-specific environmental contribution to phenotypic correlations. *C*_GCP=_ *r*_G ×_ 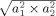; *C*_ECP =_ *r*_E ×_ 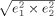, where the *a*^2^ and *e*^2^ values estimated from bivariate Cholesky decomposition models were very similar to those in Table 3.

## Discussion

This study in lean, healthy Chinese adolescents has demonstrated that PFM is inversely associated with hip geometry (CSA and SM), as well as with BA and BMC, in both genders, after accounting for body weight (mechanical-loading effect). The inverse effect of PFM on BMC mainly focuses on the total hip bone rather than the lumbar spine bone. Such relationships did not vary substantially by Tanner stage. We report for the first time on the heritability of CSA and SM in both genders, which ranged from 64% to 77% in adolescent twins. We also observed that the negative PFM-bone correlations in both genders were contributed by both shared genes and individual-specific environmental factors. Of note, in this study, BMC, with the adjustment of BA, height, and weight, was used as the main outcome instead of areal BMD (aBMD) because aBMD may be an inappropriate marker for assessing bone status in growing children,(26,27) especially during adolescence. However, we also conducted analyses using aBMD as the outcome that yielded similar results to those using BMC (data not shown).

Epidemiologic studies exploring the effects of fat mass or adiposity on adolescent bone health, most of which have limited sample size (*n* < 400),(11,13–18) have yielded conflicting results, ranging from protective effects,(11,12,18) to no effects,(13) to detrimental effects.(14–17) It is possible that these discrepancies in previous studies may due in part to such factors as differences in age, gender, bone phenotype, and study design. Another important explanation for these discrepancies is that different authors have chosen different ways to account for the confounding of mechanical-loading effect in their studies. For example, some authors presented unadjusted data,(11) whereas others presented adjusted data for lean mass only.(12,13,15,18) Our study, together with some others,(16,17) has adjusted the full mechanical-loading effect by including body weight in the regression models. We observed inverse relationships between PFM and bone parameters, which is consistent with findings from previous studies in adolescents in New Zealand,(16) in adolescent females in the United States,(14,15) and in adolescent females in Canada.(17) These consistent findings across multiple populations raise the possibility that this may be a general property of human biology.

Our study suggested that the PFM-bone relationship may vary by skeletal regions, for which PFM was associated with BMC at the hip (total hip) but not at the lumbar spine region in both genders. Notably, the quantity of cortical bone at the hip region is much higher than that at the lumbar spine region. A previous study by Pollock and colleagues also reported that areas consisting predominantly of cortical bone were affected more than areas consisting predominantly of trabecular bone by PFM.(14) These findings raised the possibility that PFM may have a differential effect on cortical versus trabecular bones. However, the underlying biologic mechanisms are not yet known and need additional research.

We observed that the magnitude of the inverse PFM-bone relationships was greater in males than in females (Fig. 2). Such gender-specific associations have been reported previously.(15,28) For example, Ackerman and colleagues suggested that BMC was lower in children with higher FM for a given sex and weight, which was more pronounced in pubertal boys.(28) Although the underlying mechanisms remain unclear, one possible explanation for the gender-specific effect is that males have a higher proportion of visceral fat than females.(29) Previous studies showed that visceral fat was associated with a higher risk of metabolic syndrome than subcutaneous fat.(30) Visceral fat also was associated with increased levels of interleukin 6 (IL-6),(31) which may be involved in bone loss and resorption.(32) A recent study has found that visceral fat is inversely associated with the structure and strength of bone.(33) Subcutaneous fat, in contrast, is positively associated with bone structure and strength.(33) Further studies are needed to investigate the molecular and functional differences of visceral and subcutaneous adipocytes and how they interact with bone.

Puberty is a time of great fluctuations in body composition and bone growth. We found no significant interaction between Tanner stage and PFM on bone parameters in our population. However, we found that in females, PFM and BMC tended to be negatively related in Tanner stages II through IV but not in Tanner stage V. This finding needs to be confirmed in a future study given the limited sample size and statistical power of this study (*n* = 103 in Tanner stage V).

It has been suggested that hip geometry is an important factor for subsequent hip fracture.(19) Studies on the relationship between fat mass and hip geometry, especially SM (an estimate of bone bending strength), might provide some additional insight into the prevention of hip fractures later in life. Petit and colleagues demonstrated that proximal femur bone geometry was adapted to lean mass rather than to fat mass in overweight children and adolescents.(13) A longitudinal study by the same investigators demonstrated that females in the “weight gain” group had a lower bone strength index (= SM/height) relative to body weight,(34) which is consistent with our findings on hip geometry. However, in their longitudinal study, Petit and colleagues reported that adiposity had a positive effect on aBMD, which does not agree with our findings. This may be due in part to the fact that they did not use normalized aBMD for body weight (as they did for bone strength index) to control the positive weight-bearing effects, whereas in our study, body weight was adjusted throughout.

Consistent with previous reports,(35) BA and BMC at different skeletal sites are highly heritable in our population. Very few studies have estimated the heritability of hip geometry, especially in adolescents. We, for the first time, provide estimates of the heritability of CSA and SM in Chinese adolescent twins. In our study, both CSA and SM are highly heritable in both genders, although the estimated heritability for SM (64%) appears to be lower than that for BMC. These estimates are slightly higher than those estimated from a Chinese adult family–based cohort (55% to 57%).(36) We also observed that shared genetics significantly contributed to the observed inverse PFM-bone relationships, indicating a set of genes shared by both PFM and bone parameters. These findings are consistent with a previous study in white adults(37) and may be explained by current understanding about the reciprocal differentiation of adipocytes (the main origin of adipokines) and osteoblasts, which each originate from the same mesenchymal stem cells in a mutually exclusive way.(38) This process is regulated by two key transcription factors, Runx2 [also called Cbfa((39))] and peroxisome proliferator–activated receptor γ (PPAR-γ2).(40) Thus genetic factors influencing the expression of Runx2 and/or PPAR-γ2 potentially may contribute to the inverse correlation between adiposity and bone health. Genetic association/linkage studies also have observed that polymorphisms on a set of genes, such as *insulin-like growth factor 1*,(41) *leptin receptor*,(42) and *IL-6*,(43) have common effects on both osteoporosis and fat mass (or obesity).

There are several limitations to this study. First, the cross-sectional design does not allow for determining the causal effect between fat mass and bone parameters, although it is difficult to consider reverse causation at play. Second, both fat mass and bone mass were derived from the same DXA measurements, which do not provide a means for distinguishing between cortical and trabecular bone and between subcutaneous and visceral fat, and this study did not account for the confounding effect of fat on bone measures when using DXA. Also, our hip geometry measurements are subject to certain technical limitations, including axial asymmetry of cross sections and the tissue mineralization assumption.(44) Third, although the twin design allows us to calculate the genetic influence on each phenotype and their correlations, it is possible that this twin cohort is not wholly representative of nontwin populations. However, we used a community-based twin cohort, and our previous reports demonstrated that this twin cohort was similar to the local general pediatric and adolescent populations with regard to socioeconomic characteristics, lifestyles, and anthropometric measurements.(45) Fourth, the number of female DZ twin pairs in this study was relatively small (*n* = 83), and the phenotypic correlations between PFM and bone in females are nonlinear (as shown in Fig. 2), which could limit our power to accurately estimate the genetic and environmental contributions to the mild to moderate phenotypic correlation we observed among the females in our cohort.

In summary, our study provides strong evidence that PFM has an inverse relationship with BMC, BA, and hip geometry for a given body weight in this sample of relatively lean Chinese adolescents and that the relationship was not affected substantially by Tanner stage. Both genetic and environmental factors contributed significantly to each of the bone parameters and to the inverse phenotypic correlation between PFM and the bone parameters. Continued follow-up of this cohort will provide further insight into the temporal relationship between PFM and bone health and the utility of PFM during adolescence as a predictor of bone mass, hip geometry, and fractures in later years.

## References

[1] Poirier P, Giles TD, Bray GA (2006). Obesity and cardiovascular disease: pathophysiology, evaluation, and effect of weight loss: an update of the 1997 American Heart Association Scientific Statement on Obesity and Heart Disease from the Obesity Committee of the Council on Nutrition, Physical Activity, and Metabolism. Circulation..

[2] Steinberger J, Daniels SR (2003). Obesity, insulin resistance, diabetes, and cardiovascular risk in children: an American Heart Association scientific statement from the Atherosclerosis, Hypertension, and Obesity in the Young Committee (Council on Cardiovascular Disease in the Young) and the Diabetes Committee (Council on Nutrition, Physical Activity, and Metabolism). Circulation..

[3] Hsu YH, Venners SA, Terwedow HA (2006). Relation of body composition, fat mass, and serum lipids to osteoporotic fractures and bone mineral density in Chinese men and women. Am J Clin Nutr..

[4] Zhao LJ, Jiang H, Papasian CJ (2008). Correlation of obesity and osteoporosis: effect of fat mass on the determination of osteoporosis. J Bone Miner Res..

[5] (2004). Bone Health and Osteoporosis: A Report of the Surgeron General.

[6] Ouyang F, Wang B, Arguelles LM (2007). Bone growth pattern in Chinese children and adolescents: a 6-year follow-up study provides evidence for sexual dimorphism and tracking. Archives of Osteoporosis..

[7] Forwood MR, Baxter-Jones AD, Beck TJ, Mirwald RL, Howard A, Bailey DA (2006). Physical activity and strength of the femoral neck during the adolescent growth spurt: a longitudinal analysis. Bone..

[8] Forwood MR (2001). Mechanical effects on the skeleton: are there clinical implications?. Osteoporos Int..

[9] Zhu K, Greenfield H, Zhang Q (2008). Growth and bone mineral accretion during puberty in Chinese girls: a five-year longitudinal study. J Bone Miner Res..

[10] Travison TG, Araujo AB, Esche GR, Beck TJ, McKinlay JB (2008). Lean mass and not fat mass is associated with male proximal femur strength. J Bone Miner Res..

[11] Leonard MB, Shults J, Wilson BA, Tershakovec AM, Zemel BS (2004). Obesity during childhood and adolescence augments bone mass and bone dimensions. Am J Clin Nutr..

[12] Clark EM, Ness AR, Tobias JH (2006). Adipose tissue stimulates bone growth in prepubertal children. J Clin Endocrinol Metab..

[13] Petit MA, Beck TJ, Shults J, Zemel BS, Foster BJ, Leonard MB (2005). Proximal femur bone geometry is appropriately adapted to lean mass in overweight children and adolescents. Bone..

[14] Pollock NK, Laing EM, Baile CA, Hamrick MW, Hall DB, Lewis RD (2007). Is adiposity advantageous for bone strength? A peripheral quantitative computed tomography study in late adolescent females. Am J Clin Nutr..

[15] Janicka A, Wren TA, Sanchez MM (2007). Fat mass is not beneficial to bone in adolescents and young adults. J Clin Endocrinol Metab..

[16] Goulding A, Taylor RW, Jones IE, McAuley KA, Manning PJ, Williams SM (2000). Overweight and obese children have low bone mass and area for their weight. Int J Obes Relat Metab Disord..

[17] Weiler HA, Janzen L, Green K, Grabowski J, Seshia MM, Yuen KC (2000). Percent body fat and bone mass in healthy Canadian females 10 to 19 years of age. Bone..

[18] Arabi A, Tamim H, Nabulsi M (2004). Sex differences in the effect of body-composition variables on bone mass in healthy children and adolescents. Am J Clin Nutr..

[19] Crabtree NJ, Kroger H, Martin A (2002). Improving risk assessment: hip geometry, bone mineral distribution and bone strength in hip fracture cases and controls. The EPOS study. European Prospective Osteoporosis Study. Osteoporos Int..

[20] Yu Y, Lu BS, Wang B (2007). Short sleep duration and adiposity in Chinese adolescents. Sleep..

[21] Marshall WA, Tanner JM (1969). Variations in pattern of pubertal changes in girls. Arch Dis Child..

[22] Marshall WA, Tanner JM (1970). Variations in the pattern of pubertal changes in boys. Arch Dis Child..

[23] Yoshikawa T, Turner CH, Peacock M (1994). Geometric structure of the femoral neck measured using dual-energy x-ray absorptiometry. J Bone Miner Res..

[24] Wang B, Necheles J, Ouyang F (2007). Monozygotic co-twin analyses of body composition measurements and serum lipids. Prev Med..

[25] Neale MC, Cardon LR (1992).

[26] Fewtrell MS, Gordon I, Biassoni L, Cole TJ (2005). Dual X-ray absorptiometry (DXA) of the lumbar spine in a clinical paediatric setting: does the method of size-adjustment matter?. Bone..

[27] Heaney RP (2003). Bone mineral content, not bone mineral density, is the correct bone measure for growth studies. Am J Clin Nutr..

[28] Ackerman A, Thornton JC, Wang J, Pierson RN, Horlick M (2006). Sex difference in the effect of puberty on the relationship between fat mass and bone mass in 926 healthy subjects, 6 to 18 years old. Obesity (Silver Spring)..

[29] Lemieux S, Prud'homme D, Bouchard C, Tremblay A, Despres JP (1993). Sex differences in the relation of visceral adipose tissue accumulation to total body fatness. Am J Clin Nutr..

[30] Fox CS, Massaro JM, Hoffmann U (2007). Abdominal visceral and subcutaneous adipose tissue compartments: association with metabolic risk factors in the Framingham Heart Study. Circulation..

[31] Cartier A, Lemieux I, Almeras N, Tremblay A, Bergeron J, Despres JP (2008). Visceral obesity and plasma glucose-insulin homeostasis: contributions of interleukin-6 and tumor necrosis factor-alpha in men. J Clin Endocrinol Metab..

[32] Ding C, Parameswaran V, Udayan R, Burgess J, Jones G (2008). Circulating levels of inflammatory markers predict change in bone mineral density and resorption in older adults: a longitudinal study. J Clin Endocrinol Metab..

[33] Gilsanz V, Chalfant J, Mo AO, Lee DC, Dorey FJ, Mittelman SD (2009). Reciprocal relations of subcutaneous and visceral fat to bone structure and strength. J Clin Endocrinol Metab..

[34] Petit MA, Beck TJ, Hughes JM, Lin HM, Bentley C, Lloyd T (2008). Proximal femur mechanical adaptation to weight gain in late adolescence: a six-year longitudinal study. J Bone Miner Res..

[35] Videman T, Levalahti E, Battie MC, Simonen R, Vanninen E, Kaprio J (2007). Heritability of BMD of femoral neck and lumbar spine: a multivariate twin study of Finnish men. J Bone Miner Res..

[36] Xu H, Long JR, Yang YJ, Deng FY, Deng HW (2006). Genetic determination and correlation of body weight and body mass index (BMI) and cross-sectional geometric parameters of the femoral neck. Osteoporos Int..

[37] Zhao LJ, Liu YJ, Liu PY, Hamilton J, Recker RR, Deng HW (2007). Relationship of obesity with osteoporosis. J Clin Endocrinol Metab..

[38] Hong JH, Hwang ES, McManus MT (2005). TAZ, a transcriptional modulator of mesenchymal stem cell differentiation. Science..

[39] Ducy P, Zhang R, Geoffroy V, Ridall AL, Karsenty G (1997). Osf2/Cbfa1: a transcriptional activator of osteoblast differentiation. Cell..

[40] Pei L, Tontonoz P (2004). Fat's loss is bone's gain. J Clin Invest..

[41] Rosen CJ, Ackert-Bicknell C, Beamer WG (2005). Allelic differences in a quantitative trait locus affecting insulin-like growth factor-I impact skeletal acquisition and body composition. Pediatr Nephrol..

[42] Fairbrother UL, Tanko LB, Walley AJ, Christiansen C, Froguel P, Blakemore AI (2007). Leptin receptor genotype at Gln223Arg is associated with body composition, BMD, and vertebral fracture in postmenopausal Danish women. J Bone Miner Res..

[43] Huang QY, Shen H, Deng HY (2003). Linkage and association of the CA repeat polymorphism of the IL6 gene, obesity-related phenotypes, and bone mineral density (BMD) in two independent Caucasian populations. J Hum Genet..

[44] Beck T (2003). Measuring the structural strength of bones with dual-energy X-ray absorptiometry: principles, technical limitations, and future possibilities. Osteoporos Int..

[45] Yu Y, Kumar R (2007). Age and gender specific lung function predictive equations provide similar predictions for both a twin population and a general population from age 6 through adolescence. Pediatr Pulmonol..

