# Supplementary figures and images for: Percent Fat Mass Is Inversely Associated With Bone Mass and Hip Geometry in Rural Chinese Adolescents
Source: J Bone Miner Res. 2010 Jan 29;25(7):1544–54. doi: 10.1002/jbmr.40 (PMC3153997; doi:10.1002/jbmr.40)

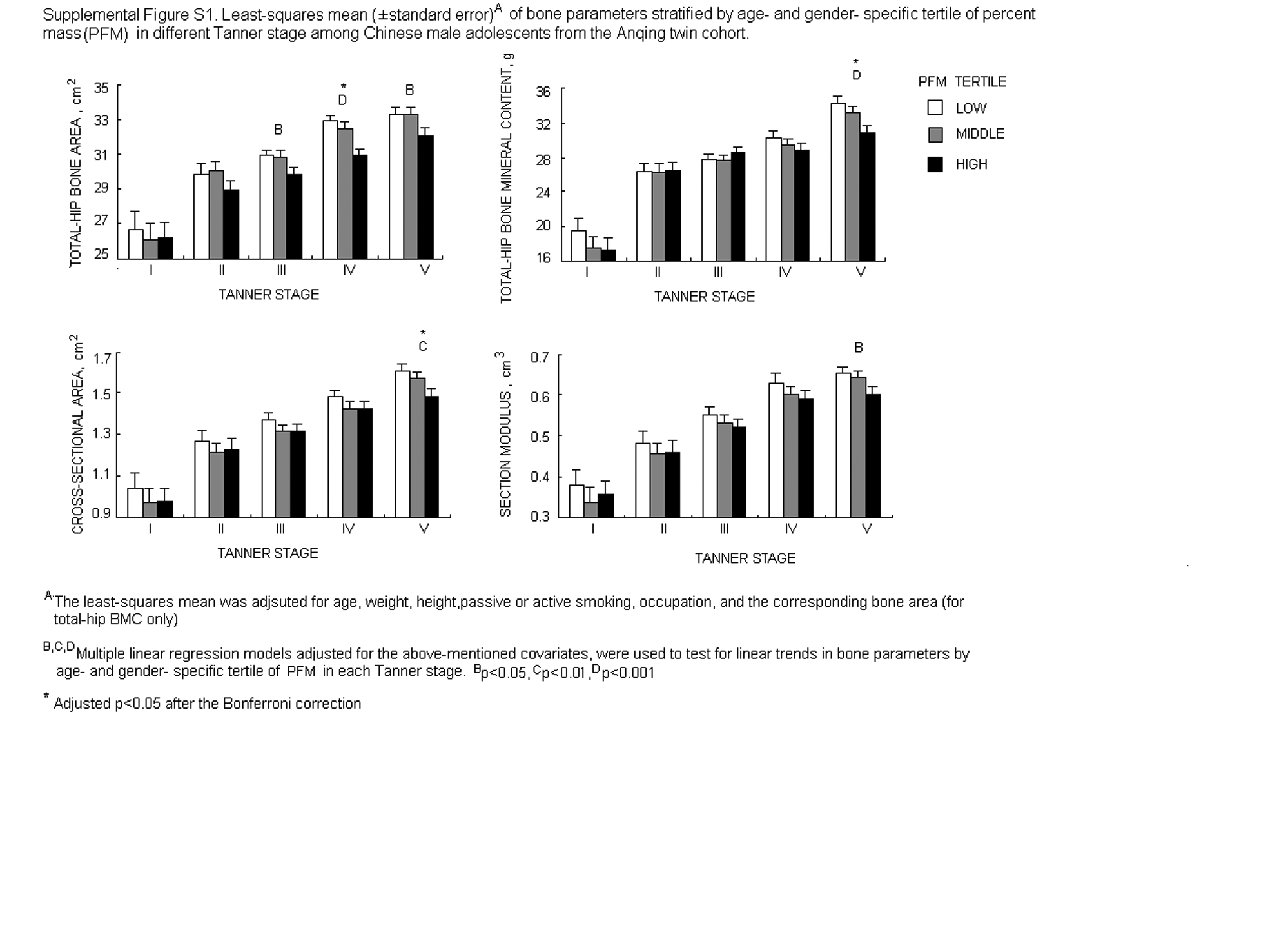

Supplement: Supplementary file 1 [file jbmr0025-1544-SD1.tif]

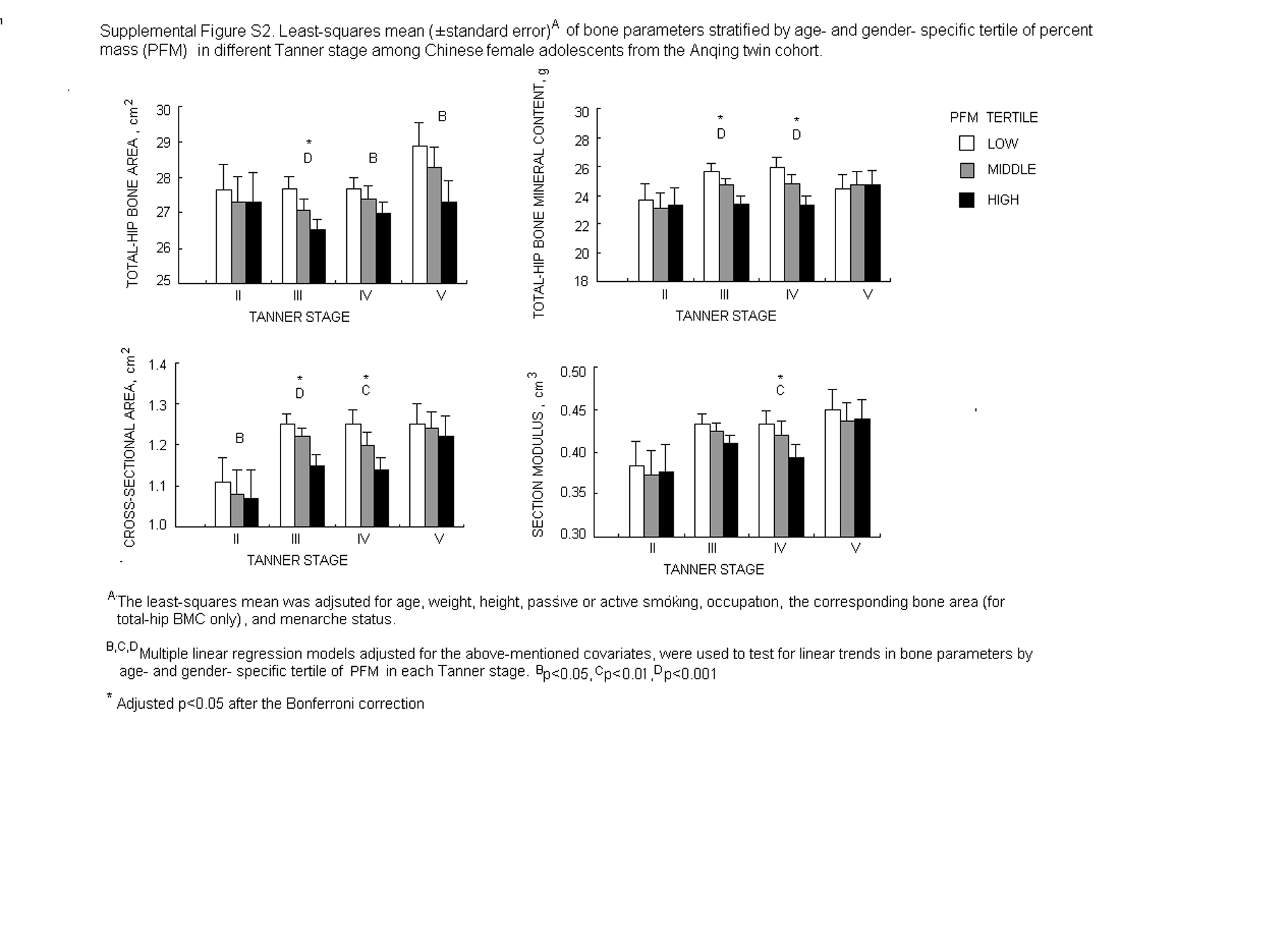

Supplement: Supplementary file 2 [file jbmr0025-1544-SD2.tif]
