# Supplementary material for: Percent Fat Mass Is Inversely Associated With Bone Mass and Hip Geometry in Rural Chinese Adolescents
Source: J Bone Miner Res. 2010 Jan 29;25(7):1544–54. doi: 10.1002/jbmr.40 (PMC3153997; doi:10.1002/jbmr.40)
Supplement: Supplementary file 3 [file jbmr0025-1544-SD3.doc]

**Online Supplemental Tables**

**Table S1. Association of bone mineral content (BMC) with hip geometry by skeletal sites in 1,404 Chinese adolescents from the** Anqing twin cohort.

| **BMC** | **Male** | | **Female** | | | | **Total** | |
| --- | --- | --- | --- | --- | --- | --- | --- | --- |
|  | **ß±se ^a^** | **ß±se ^b^** |  | **ß±Se ^a^** | **ß±Se ^b^** |  | **ß±Se ^a^** | **ß±Se ^b^** |
| **Cross-sectional area** | | | | | | | | |
| Whole-body | 0.001±0.00 ^c^ | 0.001±0.00 ^c^ | 0.001±0.00 ^c^ | | 0.001±0.00 ^c^ | | 0.001±0.00 ^c^ | 0.001±0.00 ^c^ |
| Lumbar-spine | 0.02±0.00 ^c^ | 0.02±0.00 ^c^ | 0.02±0.00 ^c^ | | 0.02±0.00 ^c^ | | 0.02±0.00 ^c^ | 0.02±0.00 ^c^ |
| Total-hip | 0.04±0.00 ^c^ | 0.04±0.00 ^c^ | 0.04±0.00 ^c^ | | 0.04±0.00 ^c^ | | 0.04±0.00 ^c^ | 0.04±0.00 ^c^ |
| **Section Modulus** | | | | | | | | |
| Whole-body | 0.0003±0.00 ^c^ | 0.0003+0.00 ^c^ | 0.0003±0.00 ^c^ | | 0.0003+0.00 ^c^ | | 0.0004±0.00 ^c^ | 0.0004+0.00 ^c^ |
| Lumbar-spine | 0.01±0.00 ^c^ | 0.01+0.00 ^c^ | 0.01±0.00 ^c^ | | 0.01+0.00 ^c^ | | 0.01±0.00 ^c^ | 0.01+0.00 ^c^ |
| Total-hip | 0.02±0.00 ^c^ | 0.02+0.00 ^c^ | 0.01±0.00 ^c^ | | 0.01+0.00 ^c^ | | 0.01±0.00 ^c^ | 0.01+0.00 ^c^ |

^a^ The model adjusted for age, Tanner stage, weight, height, menarche status(for females only), physical activity, passive or active smoking, occupation, gender (for the total population only), and the responding bone area (for BMC only).

^b^ The model adjusted all the covariates listed above, plus age- and gender- specific tertile of percent fat mass.

^c^ p<0.0001，adjusted p<0.05 after the Bonferroni correction.

**Table S2. Chi-square test of model-fitting for bone parameters ^a^ and percent fat mass (PFM) ^a^**, in 590 same-sex Chinese twins from the Anqing twin cohort.

|  | **WB-BA** | **L2l4-BA** | **TH-BA** | **WB-BMC** | **L2L4-BMC** | **TH-BMC** | **CSA** | **SM** | **PFM** |
| --- | --- | --- | --- | --- | --- | --- | --- | --- | --- |
| **Male** | | | | | | | | | |
| **AE vs ACE** | 0.00 | 0.24 | 0.04 | 0.00 | 0.00 | 0.00 | 0.00 | 0.04 | 1.77 |
| **CE vs ACE** | 56.34 ^d^ | 33.72 ^d^ | 27.29 ^d^ | 75.32 ^d^ | 92.44 ^d^ | 55.18 ^d^ | 36.42 ^d^ | 14.9 ^d^ | 47.24 ^d^ |
| **Female** | | | | | | | | | |
| **AE vs ACE** | 0.05 | 1.26 | 3.54 | 2.40 | 0.36 | 0.00 | 0.927 | 2.42 | 0.86 |
| **CE vs ACE** | 23.32 ^d^ | 8.16 ^c^ | 21.47 ^d^ | 23.07 ^d^ | 21.29 ^d^ | 54.91 ^d^ | 22.56 ^d^ | 4.45 ^b^ | 20.71 ^d^ |

WB-BA: whole-body less head bone area (BA); L2L4-BA: lumbar-spine BA, TH-BA: total-hip BA; WB-BMC: whole-body less head bone mineral content (BMC); L2L4-BMC: lumbar-spine BMC, TH-BMC: total-hip BMC. CSA: cross-sectional area; PFM: percent fat mass.

^a^ Adjusted for age, body weight, height, menarche status(for females only), passive or active smoking, occupation and the corresponding bone area (for total-hip BMC only).

**Δχ^2^** was shown in the table. ^b,c.d^ **^Δ^**χ ^2^ >3.84(p<0.05) indicate that the nested model (e.g. CE model) provides a worse fit to the data than the full ACE model., ^b^ p<0.05, ^c^ p<0.01, ^d^ p<0.001
